# Supplementary figures and images for: Review and Evaluation of Ostertagia ostertagi Antibody ELISA for Application on Serum Samples in First Season Grazing Calves
Source: Animals (Basel). 2023 Jul 6;13(13):2226. doi: 10.3390/ani13132226 (PMC10339862; doi:10.3390/ani13132226)

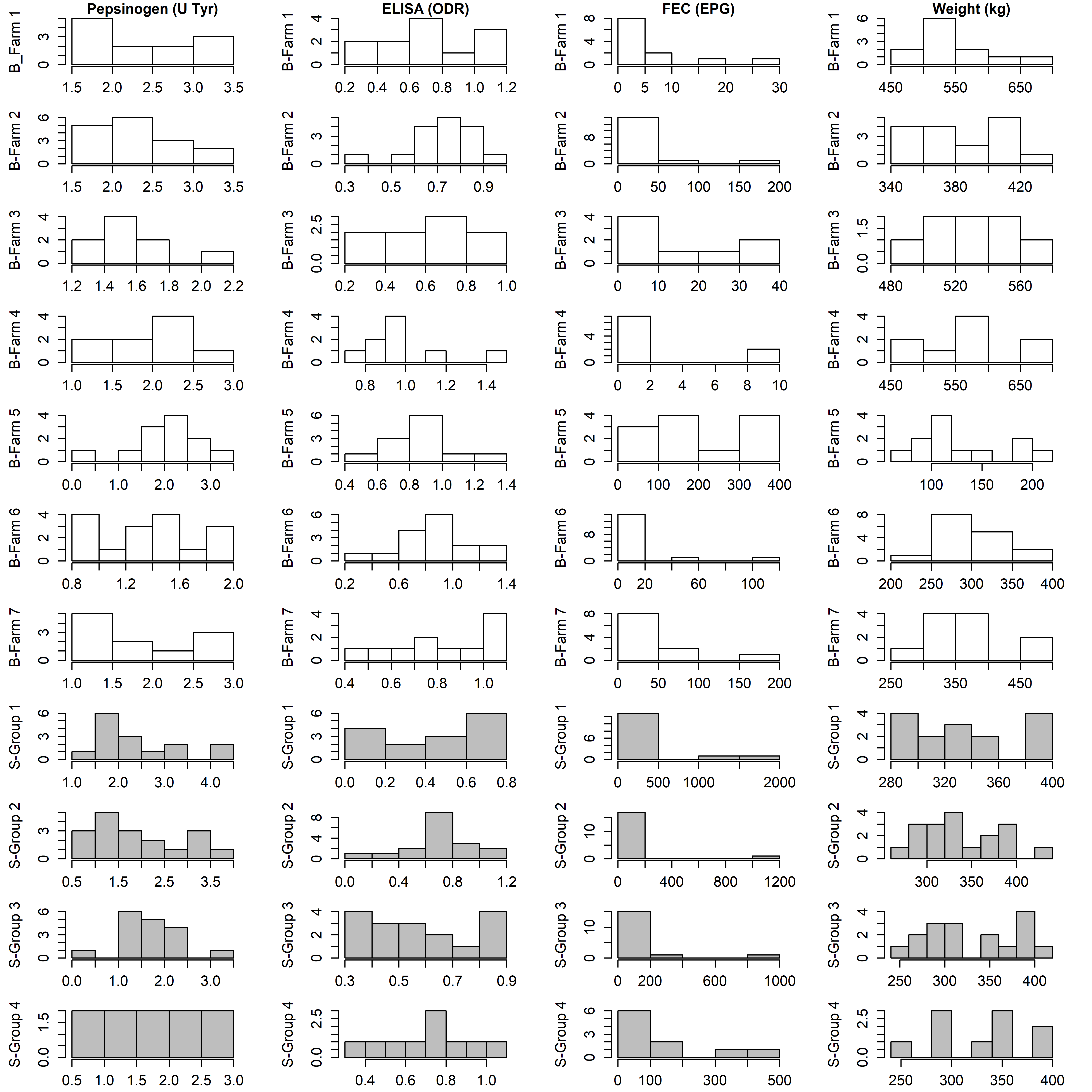

Supplement: Supplementary file 1 [file animals-13-02226-s001.zip › animals-2464182-supplementary.png]
